# Binding and inactivation of human coronaviruses, including SARS-CoV-2, onto purified clinoptilolite-tuff

**DOI:** 10.1038/s41598-023-31744-z

**Published:** 2023-03-22

**Authors:** S. Nizet, J. Rieger, A. Sarabi, G. Lajtai, K. Zatloukal, C. Tschegg

**Affiliations:** 1 Glock Health, Science and Research GmbH, Hausfeldstrasse 17, 2232 Deutsch-Wagram, Austria; 2 Diagnostic and Research Institute of Pathology, Medical University Graz, Neue Stiftingtalstrasse 6, 8010 Graz, Austria; 3 Wernberg, Austria

**Keywords:** SARS-CoV-2, Viral epidemiology, Viral transmission

## Abstract

The current COVID19 pandemic is caused by a positive-sense single-stranded RNA virus, which presents high mutational rates. The development of effective therapeutics and mitigation strategies using vaccination or therapeutic antibodies faces serious challenges because of the regular emergence of immune escape variants of the virus. An efficient approach would involve the use of an agent to non-specifically limit or block viruses contacting the mucosae and therefore entering the body. Here, we investigated the ability of a micronized purified clinoptilolite-tuff to bind and neutralize different viruses from the *Coronaviridae* family. Using plaque assay, RT-qPCR and immunostaining, the adsorption and inactivation of the seasonal human coronavirus HCoV-229E and of 2 SARS-CoV-2 variants were demonstrated. The resulting data suggest that purified clinoptilolite-tuff could be used as an ingredient in new medical devices and/or pharmaceuticals to prevent or mitigate SARS-CoV-2 dissemination.

## Introduction

Coronaviruses are enveloped viruses with a positive-sense single-stranded RNA genome, which cause respiratory or gastrointestinal diseases in birds and mammals. The recent emergence of human-pathogenic variants with a high potential that can lead to a global pandemic, exemplified by SARS-CoV-2, is a serious warning for the medical community and new strains and variants will likely continue to emerge in the future^1^.

SARS-CoV-2 is an airborne viral pathogen and the upper respiratory tract and specifically the nasopharynx are considered as the very first target sites of SARS-CoV-2 infection^2^. This is supported by the fact that high virus loads were measured in nasopharyngal swabs soon after symptoms onset^3^. Moreover, co-expression of the ACE2, TMPRSS2 receptors and furin, which are key actors for an efficient cellular entry of SARS-CoV-2 for infection, has been demonstrated in the ciliated nasal cells as well as some intestinal cells^4^. However, during the course of disease, cells of the lower respiratory track can also get infected, which may lead to the development of severe pneumonia and systemic disease^5^.

Due to limited therapeutic options, the main strategy against the pandemic is based on the systemic immunization with vaccines. However, this strategy was faced by substantial challenges in achieving high vaccination rates within a population, and by heterogeneity in vaccine response^5^. Rising concerns over the appearance of new variants harboring mutations in the viral spike protein, which may provide some form of immune escape from the acquired immunity, call for the implementation of multiple, complementary strategies to control the pandemic^6,7^.

In this regard, blocking the virus at the entry site where mucociliary clearance and local immune mechanisms are defective or have reached their limits could represent an efficient approach to deal with high virus loads. In this context, several nasal sprays have already been brought to the market to aid limiting respiratory infections or allergic reactions^8,9^. Some may even be used prophylactically^10^.

An ideal antiviral substance would bind and/or inactivate all coronavirus variants independently of their mutations. Binding of viruses by mineral substances has already been demonstrated using sand and soil beds^11,12^, diatomaceous earth^13^, diosmectite^14^ and clays^15,16^. Even though the binding mechanisms are unspecific, sorption may be so strong that only an insignificant proportion of the bound virus can effectively be desorbed^15^. One such potent mineral, called zeolite, has demonstrated interesting properties to fight viral diseases in animals^17,18^. Powdered zeolite-rocks with a high clinoptilolite content, named clinoptilolite-tuff, showed antiviral properties against adenoviruses, enteroviruses and herpes virus^19^.

Such clinoptilolite-tuffs, which are extracted from quarries, are naturally loaded with heavy metals and may represent a health hazard when used orally or introduced in the body over prolonged periods^20^. For this reason, we developed a purifying process to obtain a clean fine powder, named purified clinoptilolite-tuff (PCT), which presents a strong binding capacity^21,22^ and was shown to be safe through oral administration in a human trial^23^ and a mouse colitis model^24^, and by topical application in a wound healing study^25^. In the mouse colitis model, PCT was trapped in the intestinal mucus layer and wasn’t internalized by gut epithelial cells^24^.

In the present study, the ability of PCT to bind and inactivate viruses of the *Coronaviridae* family and, more specifically, several variants of SARS-CoV-2, was investigated. Binding was demonstrated indirectly by the reduction of virus copy numbers and mitigated infectivity toward a permissive cell line and directly by scanning electron microscopy (SEM) upon incubation of virions with PCT. Binding and inactivation were further established by plaque assay. By quantifying the virions using RT-qPCR directly after incubation with PCT and after amplification in Vero E6 cells, both binding and inactivation of different SARS-CoV-2 variants by PCT could clearly be demonstrated. Our results suggest that PCT in a liquid or gel suspension may represent a natural, safe and effective candidate for the development of a medical device destined to reduce SARS-CoV-2 infectivity and virus spread in patients or as an adjunct in the prophylaxis of the current SARS-CoV-2 pandemic.

## Results

### Binding of human coronavirus HCoV-229E to PCT

Natural zeolites are known to bind and inactivate various bacteria and bacterial products as well as viruses^15,21,26,27^. We hypothesized that PCT can also bind members of the *Coronaviridae* family and first used the less pathogenic strain HCoV-229E.

Interaction of HCoV-229E virions with PCT was investigated indirectly by counting the plaque-forming units (PFU) remaining in the supernatant upon incubation with 0%, 0.01% and 0.1% PCT.

**Figure 1:**
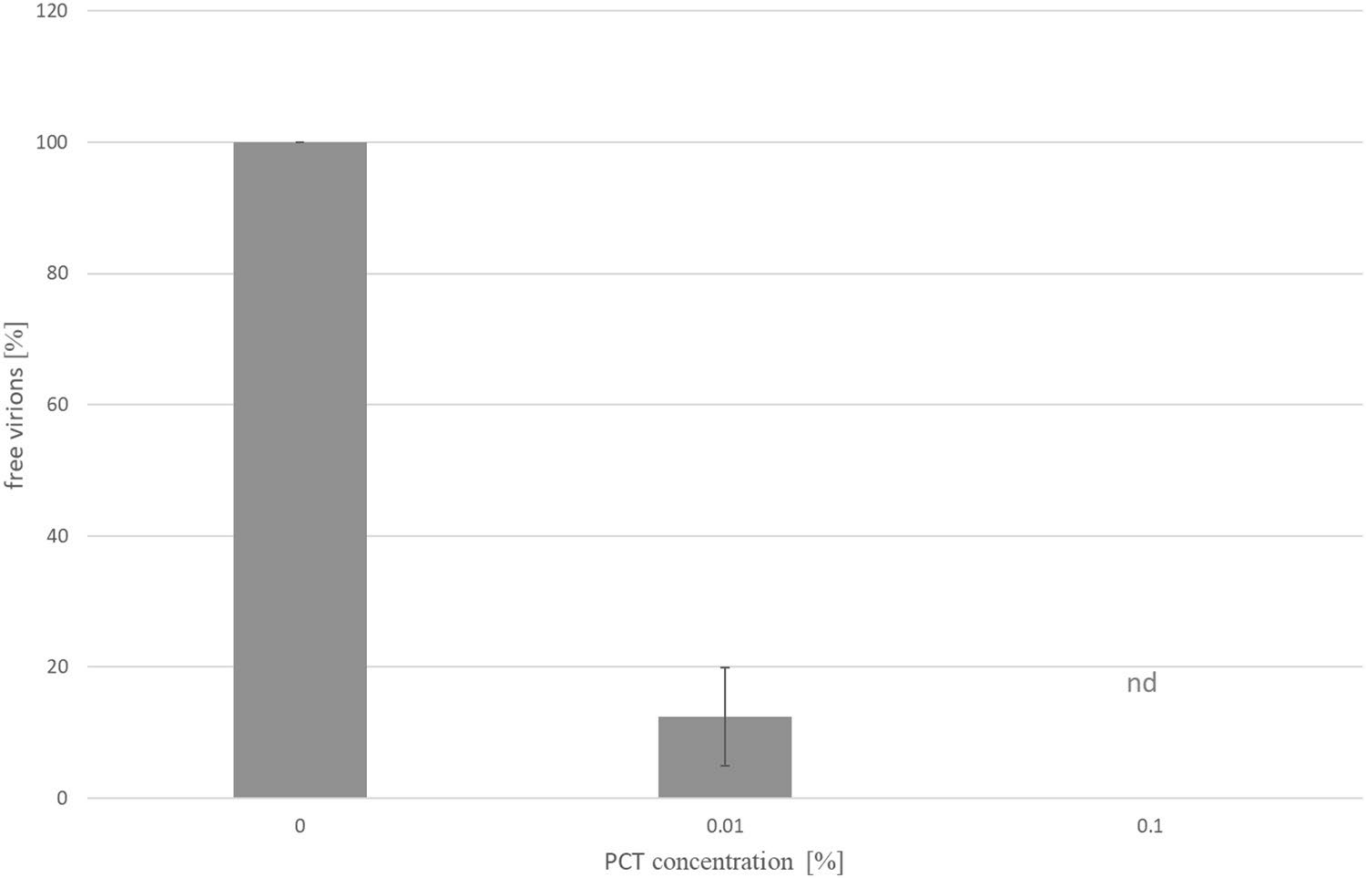
Effect of PCT on HCoV-229E coronavirus as measured by plaque reduction assay. Increasing concentrations of PCT were incubated with a suspension of HCoV-229E at 16,000 PFU per ml for 1 h at room temperature and the free virus concentration was assessed by plaque assay. Data from 3 independent experiments normalized relative to the “0% PCT” sample. Means and S.D. are shown.

Addition of 0.01% PCT to a HCoV-229E suspension containing 16,000 PFU per ml resulted in an 87% reduction of the virus concentration (Fig. 1). No PFU was detected when the PCT concentration was increased to 0.1% (Fig. 1).

Next, direct binding of HCoV-229E virions onto PCT was demonstrated by scanning electron microscopy. The virus suspension was incubated with PCT before fixation and the particles were then retained onto a filter membrane. After extensive washing, the filter was dried, metal coated and observed by SEM.

**Figure 2:**
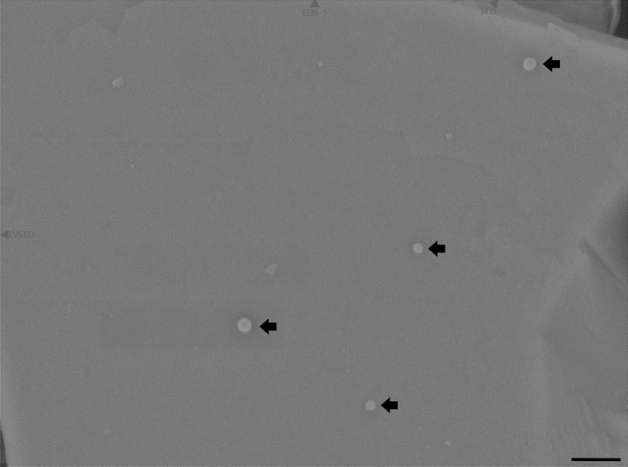
Scanning electron microscopy observation of HCoV-229E virus binding onto PCT. PCT was incubated with HCoV-229E, fixed with paraformaldehyde, filtered through a 0.05 µm filter membrane, washed, air dried, metal coated and observed in SEM. Images were taken using the secondary electron detector at 20,000-fold magnification. The scale bar represents 0.5 µm. Arrows show viruses on the surface of a particle of PCT.

Virus particles were observed on the surface of PCT microparticles (Fig. 2). By contrast, no such virus particles were visible on mock-incubated PCT.

### Saturation curve of HCoV-229E inactivation by PCT

To better characterize the interaction of PCT with HCoV-229E and in particular to quantify the interaction capacity, decreasing amounts of the virus were incubated with a constant concentration of PCT, pelleted by centrifugation, and the free viral particles present in the supernatant were analyzed by plaque reduction assay.

**Figure 3:**
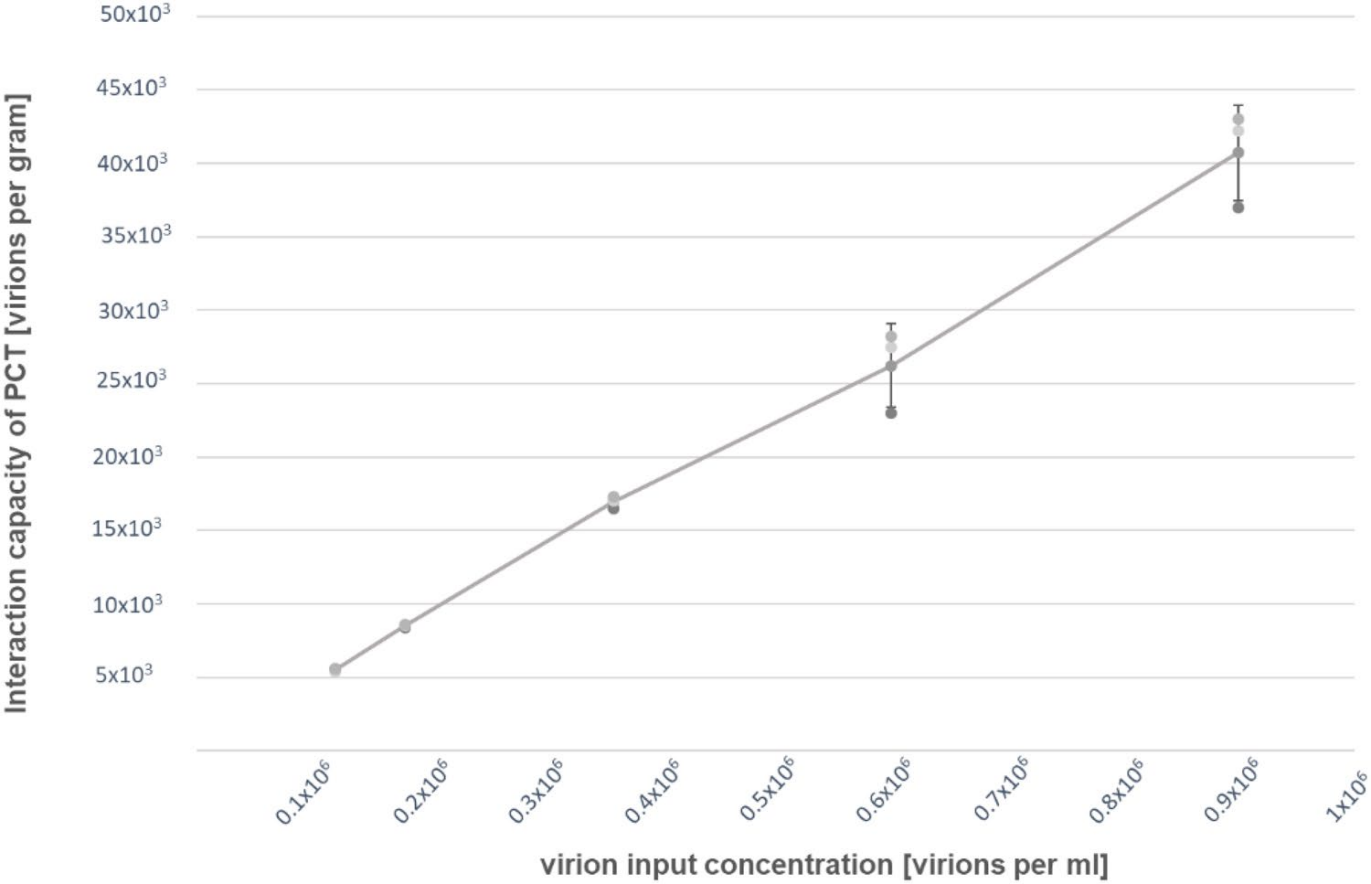
Saturation curve of HCoV-229E interaction with PCT. A constant amount of PCT (0.002%) was added to the indicated virus concentration and incubated for 1 h at 37 °C. After centrifugation, the remaining free virions in the supernatant were quantified by plaque reduction assay and the binding and/or inactivation capacity of one gram PCT was calculated. Data from 3 independent experiments carried on in duplicate. Means and S.D. are shown.

Interaction with PCT followed a linear relationship with the input virus concentration (Fig. 3). In our hands, no saturation could be reached. Nonetheless, the observed maximal interaction capacity was greater than 40.7 ± 3.3 billion infectious viral particles per gram PCT.

### Binding and inactivation of human coronavirus SARS-CoV-2 by PCT

**Figure 4:**
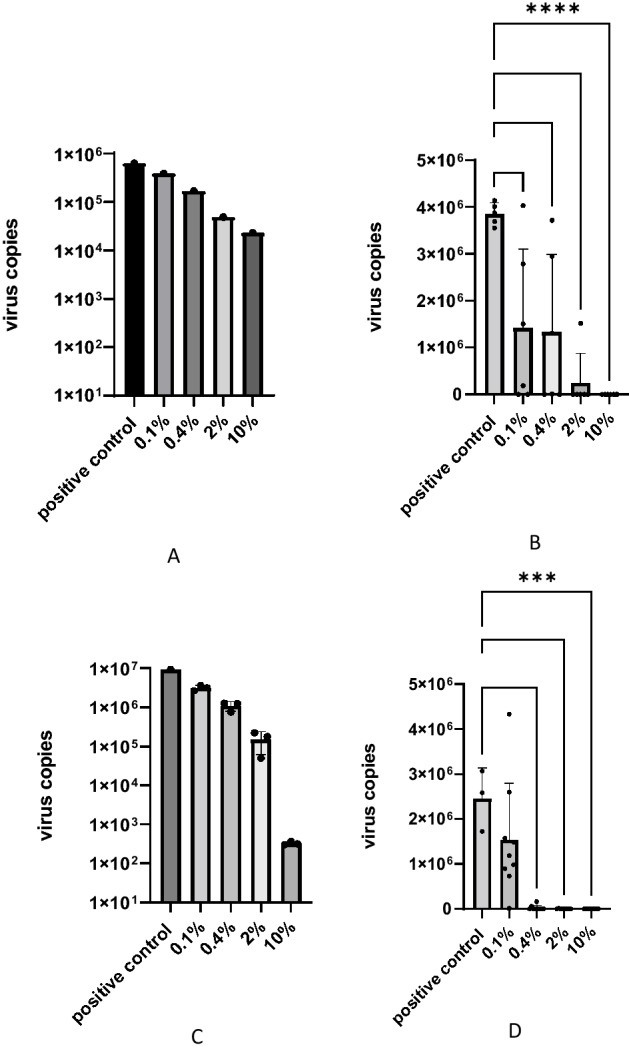
SARS-CoV-2 virus adsorption and inactivation by PCT. A fixed concentration of a SARS-CoV-2 suspension was incubated with increasing concentrations of PCT and the unbound virions in the supernatant were quantified by RT-qPCR either after incubation with PCT (**A**,** C**) or after subsequent infection and further cultivation of Vero E6 cells (**B**,** D**). The experiments were performed with the SARS-CoV-2 Wuhan strain (**A**,** B**) and the SARS-CoV-2 Omicron B.1.1.529 variant (**C**,** D**). Individual values are displayed as dots, mean values as bars and standard deviations as error bars. All datasets in triplicate *p* > 0.05 (not significant; ns); *p* ≤ 0.05 (*); *p* ≤ 0.01 (**); *p* ≤ 0.001 (***).

Knowing that PCT can bind high amounts of a seasonal coronavirus in vitro, we next tested if this holds true for the highly pathologic SARS-CoV-2 as well. To this end, different concentrations of PCT were incubated with a constant concentration of SARS-CoV-2 (Wuhan strain) and the unbound virus was quantified by RT-qPCR. A 27-fold reduction of the free virion concentration was observed upon incubation of the SARS-CoV-2 Wuhan strain with 10% PCT (Fig. 4A). Further dilutions of PCT revealed a linear inverse relationship between the PCT concentration and the concentration of free SARS-CoV-2 virions.

The next question was whether the incubation of SARS-CoV-2 with PCT reduces infectivity by adsorption or whether there is an additional virus neutralizing effect. This was tested by infecting Vero E6 cells with SARS-CoV-2 after incubation with PCT. Virus infection and replication were determined by measuring the number of virus particles in the cell culture supernatant after infection of Vero E6 cells and further cultivation for 48 h. Cells infected with untreated virus led to 3.85 × 10^6^ virus copies per µl virus stock after 48 h of infection and there was a slight reduction in virus copy numbers (1.4 × 10^6^ copies per µl stock) after infection with SARS-CoV-2 Wuhan treated with 0.1% PCT (Fig. 4B), whereas infection with virus treated with 10% PCT led to values near the limit of detection in the RT-qPCR analysis with 0.5 virus copies per µl virus stock (Fig. 4B). By comparing the Ct values from the virus binding assay (up to 32-fold reduction) with those from the unbound virus in the inactivation assay (up to 2 × 10^7^-fold reduction), a net neutralizing activity of approximately 6.25 × 10^5^-fold was calculated.

**Figure 5:**
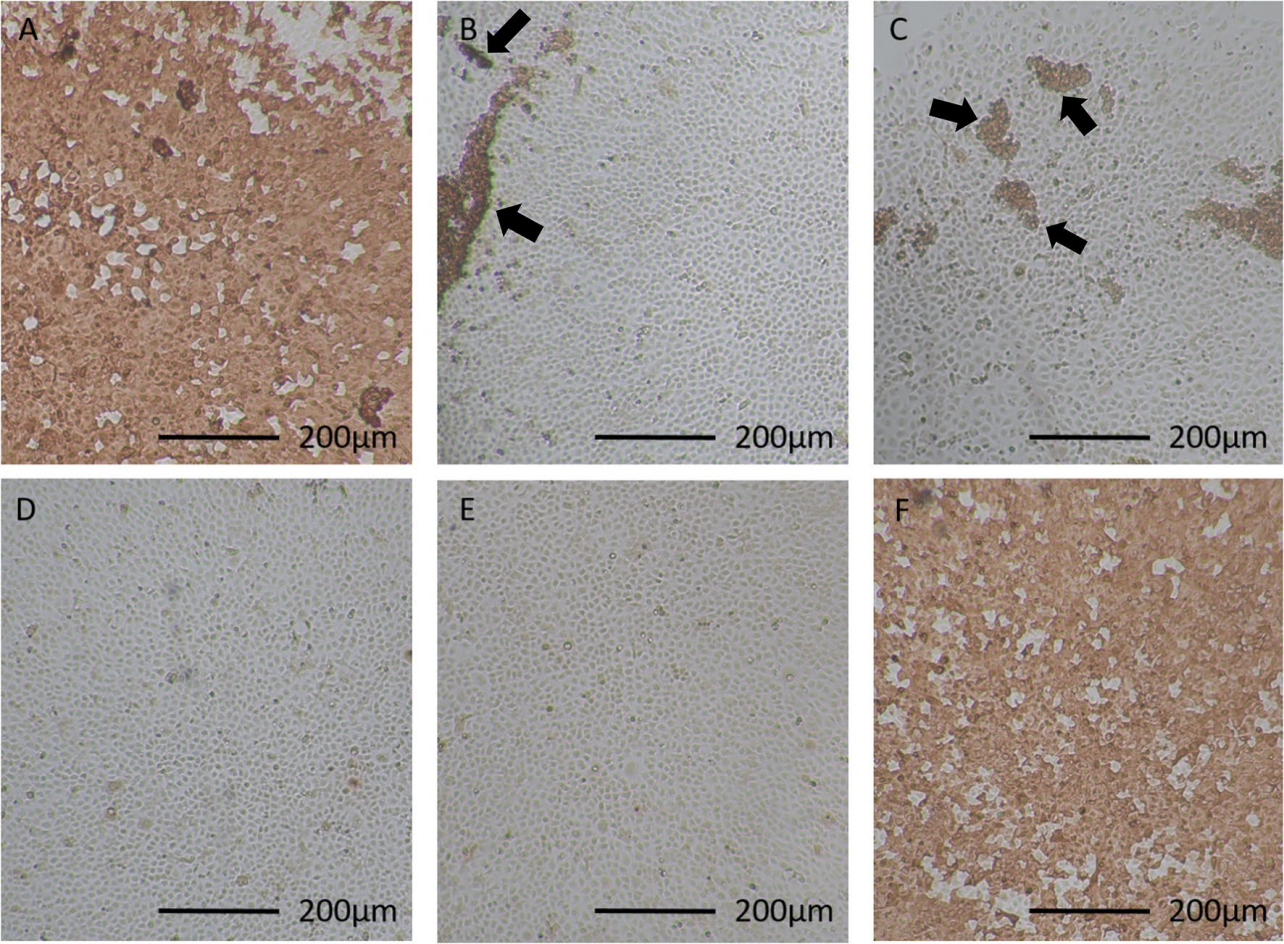
Immunohistochemical staining of the cells used for virus copy number analysis in the experiment shown in Fig. 4D. Infected cells are stained in brown. (**A**) cells infected with 0.1% PCT-treated virus; (**B**) cells infected with 0.4% PCT-treated virus; (**C**) cells infected with 2% PCT-treated virus; (**D**) cells infected with 10% PCT-treated virus; (**E**) negative control without virus; (**F**) positive control made of cells infected with untreated virus. Arrows show examples of positively stained cell groups. Photomicrographs were taken using Jenoptik GRYPHAX software Version 2.2.0.1234 (https://www.jenoptik.com/products/cameras-and-imaging-modules/microscope-cameras/progres-gryphax-usb-30-microscope-workstation).

Since a virus-neutralizing activity of PCT is most likely related to a physical–chemical interaction with the virus, it is expected to have the same effect with different SARS-CoV-2 variants. We therefore tested adsorption and inactivation of the SARS-CoV-2 Omicron B.1.1.529 variant, which was at the time of the study the predominant variant in circulation. Results of the adsorption experiment showed decreased virus copies of the Omicron B.1.1.529 variant upon incubation with PCT, the extent of which was dependent on the PCT concentration (Fig. 4C). In comparison to the Wuhan strain, the Omicron B.1.1.529 variant showed more pronounced adsorption to PCT, particularly at high PCT concentrations (compare Fig. 4A and C). Like in the experimental series with the SARS-CoV-2 Wuhan strain, the infectivity of the Omicron B.1.1.529 variant was tested after incubation with PCT in a virus inactivation assay. The results showed that inactivation of the omicron B.1.1.529 variant took place in a dose-dependent manner. With 10% PCT, the virus concentration in cell culture supernatant reached a 1.81 × 10^6^-fold reduction compared to the infection with untreated virus (Fig. 4D). This effect was not only due to virus adsorption but also to the virus neutralizing activity of PCT. This was even more evident with 2% PCT, where adsorption alone was responsible for an 89.5-fold decrease in virus copies (Fig. 4C), whereas the reduction in virus copies after infection of cells reached 4.87 × 10^4^-fold in the inactivation experiment (Fig. 4D). The virus neutralizing activity for the Omicron B.1.1.529 variant was further investigated by using immunohistochemical labeling of the infected cells of the experiment shown in Fig. 4. This independent readout confirmed the results from the RT-qPCR analysis (Fig. 5). Virus treated with 2% and 10% PCT inhibited virus replication below the limit of detection by immunohistochemistry whereas almost all cells were positively stained for SARS-CoV-2 upon pre-treatment with 0.4% or 0.1% PCT.

### Metabolic interference of PCT in cell culture

**Figure 6:**
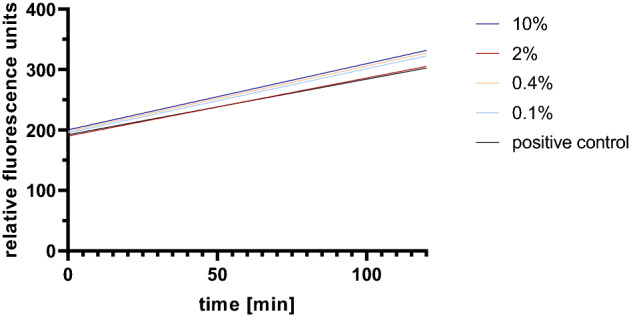
Metabolic interference assay performed on Vero E6 cells with different concentrations of PCT (0.1%, 0.4%, 2%, 10%) over a time period of 48 h. The slope reflects the change in resazurin fluorescence measured over 2 h as indicator of metabolic activity. The experiment was performed in triplicates and the mean values are displayed.

In order to exclude that the virus neutralizing effect of PCT might be the consequence of a metabolic interference of the substance with the cells, which might impair virus replication in a non-specific manner, a resazurin-based metabolic interference assay was performed. Using the same experimental conditions as those applied for the virus inactivation assay (same concentrations and incubation times) it was found that concentrations up to 10% PCT had no major effect on the metabolic activity of Vero E6 cells (Fig. 6).

## Discussion

The current COVID-19 pandemic is caused by the enveloped, positive single‐stranded RNA virus SARS-CoV-2 belonging to the Betacoronavirus genus. The heavy burden caused by this virus resulted in intense efforts worldwide aimed at developing various strategies to fight the infection and leading to a variety of measures such as obligatory wearing of face masks, social distancing or even lock downs as well as vaccination strategies and administration of oral antivirals.

Another complementary strategy is based on inactivating viral particles by administration of neutralizing monoclonal antibodies^28^. However, because SARS-CoV-2 mutates fast^29^ and due to the development of herd immunity through natural immunity and vaccines, it is reasonable to expect an acceleration of the emergence of new variants through accentuated selection pressure on the virus^7,30^. The high specificity of monoclonal antibody epitope recognition would then lead to a cat-and-mouse chase for the best therapy and the never-ending development of new variants and more adapted neutralizing antibodies^31^.

In order to develop a material capable of widely neutralizing the increasing number of SARS-CoV-2 variants, it is important to find a substance which interacts with the virus in a relatively non-specific manner. In this regard, a few natural substances are currently investigated^8,14,32,33^.

One of these natural substances, clinoptilolite-tuff, a type of zeolitic material, has already demonstrated excellent binding capabilities toward various substrates^21,22,34^ and therefore is a promising binding material.

In this work, binding of purified clinoptilolite-tuff (PCT) to coronavirus was first tested with the HCoV-229E strain, which is a cause of common cold and belongs to the Alphacoronavirus genus. Incubating a suspension of HCoV-229E virus with PCT and analyzing the unbound viral particles in the supernatant showed that nearly all HCoV-229E virions lost their infectivity in a dose-dependent manner onto interaction with PCT within 1 h as determined by the decrease of plaque forming units (PFU). Although the mode of action is yet unclear, direct binding of HCoV-229E particles was demonstrated by SEM (Fig. 2). These results are in line with previous publications: already in 1984, Lipson and Stotzky showed that kaolinite and montmorillonite, 2 clays closely related to zeolite, were able to retain reoviruses in soil^16^. Almost 3 decades later, using the same minerals, Chrysikopoulos et al. demonstrated the attachment of 2 bacteriophages^35^. These results were confirmed recently by Poeta et al., who showed trapping of SARS-CoV-2 particles by diosmectite, another name for montmorillonite^14^. In 1998, Clark et al. demonstrated the adsorption of rotavirus to kaolinite and mordenite in vitro, two alumosilicates closely related to zeolite^15^. Finally, Grce showed that micronized clinoptilolite-zeolite can bind human adenovirus, herpes simplex and enteroviruses *in vitro*^19^. Altogether, these results indicate that alumosilicate minerals, and PCT in particular, possess general binding properties towards many different viruses.

Based on the results showing inhibition of infection by HCoV-229E in cell culture, further experiments were performed with SARS-CoV-2. Direct binding of SARS-CoV-2 onto PCT was investigated by using RT-qPCR to quantify virus particles before and after interaction with PCT. A potential additional reduction of infectivity was further tested by incubating the unbound virions with permissive cells .This so-called inactivation experiment allowed distinction between reduction of infectivity by binding to PCT or by another mechanism. These experiments on the one hand demonstrated a dose-dependent adsorption of SARS-CoV-2 to PCT and, on the other hand, inactivation of unbound virus, which could not be pelleted together with PCT by centrifugation after pre-incubation. Although the centrifugation does not exclude the presence of small PCT particles in the supernatant containing unbound virus, the marked difference in virus infectivity as seen after incubation with 2% or 10% PCT (Fig. 4) cannot be explained by adsorption alone and suggests additional virus neutralizing activity of PCT. This effect was shown similarly for two SARS-CoV-2 variants, namely the original Wuhan and the Omicron B.1.1.529 variants. The difference between both strains at 0.4% PCT in the metabolic interference assay may be explained by a slight difference in cell permissivity. This is evidenced by a recent study, which showed different infection efficacies of Omicron B.1.1.529 compared to other variants^36^.

Since reduction of in vitro infectivity of *Coronaviridiae* was shown with the Alphacoronavirus and the two Betacoronavirus variants Wuhan and Omicron, which use different receptors to infect cells^37^, a general, relatively unspecific virus neutralizing effect was demonstrated. This general activity might be a major advantage over more specific anti-viral agents for emerging new virus variants. In this context it is also important to demonstrate that the virus neutralizing effect was not due to general interference with cell function. Although PCT could be safely used in vivo^24,25^ and in vitro^21^, its effect on the Vero E6 cells used in this work has not been tested previously. Therefore, we performed metabolic interference studies using a resazurin assay, which measures the cellular metabolic activity through the reduction of resazurin by the cellular mitochondrial reductase. By applying the same experimental conditions as used for the virus inactivation experiment, only minor changes in metabolic activity were observed, which suggests that the concentrations of PCT applied do not impair the metabolic activity of the cells.

In conclusion, our data suggest that PCT directly adsorbs and inactivates several members of the *Coronaviridae* family, which opens a variety of applications for local administration, such as nasal spray to mitigate virus infection in the nasopharynx or as orally administered suspension to reduce virus infectivity in the mouth and the intestinal tract.

## Materials and methods 

### Purified clinoptilolite-tuff

Purified clinoptilolite-tuff (PCT) was prepared from a high-grade raw material with low heavy metal content, sourced from an open-pit mine in the eastern Slovak Republic^20,38^. The patented purification process is based on ion exchange mechanisms of the clinoptilolite mineral, micronization and terminal heating, which results in the removal of all natural impurities and a homogeneous, very fine-grained particle size. The production process is thoroughly quality assured, meeting all required regulatory standards. The final purified product has been evaluated by independent laboratories and trials conforming the safety requirements for human consumption and efficacy. In the U.S., PCT is marketed as G-PUR®, which has been successfully applied in different therapeutic indications^22,23,39^.

Before incubation with the virus suspensions, PCT was sterilized with 70% ethanol. The alcohol was eliminated by centrifugation at 2000 × g for 10 min and PCT powder was dried for 15 min at room temperature (RT) under the laminar flow. Thereafter the PCT was suspended in serum-free medium by vortexing for 5 min and diluted to achieve the various concentrations to be tested.

### Virus strains, cell culture and coronavirus propagation

Human coronavirus HCoV-229E (VR-740) and MRC-5 cells (CCL-171) were obtained from the American Type Culture Collection (Manassas, VA, USA). MRC-5 cells were grown in Eagle MEM (PAN-Biotech, Aidenach, Germany) supplemented with 10% fetal bovine serum (FBS, Catus Biotech, Tutzing, Germany), 100 U/ml penicillin, 100 µg/ml streptomycin (AppliChem, Darmstadt, Germany), 2 mM L-Glutamine, 1 mM sodium pyruvate, and 1% non-essential amino-acids (all from PAN-Biotech, Aidenbach, Germany).

For propagation of HCoV-299E, MRC-5 cells grown until confluence in 150 cm^2^ flasks (TPP, Trasadingen, Switzerland) were infected with 5 µl of the original virus suspension from ATCC (concentration 1.7 × 10^6^ virions per ml). The supernatant was collected after 5 days at 35 °C, cleared by a 5-min centrifugation at 4000 × g, aliquoted and stored at −80 °C until further use.

Human 2019-nCoV Isolate Wuhan strain (Ref-SKU: 026 V-03883) and SARS-CoV-2 Omicron strain (SARS-CoV-2, strain hCoV-19/Netherlands/NH-EMC-1720/2021, Omicron variant, lineage B.1.1.529) were obtained from Charité-Universitätsmedizin Berlin and Erasmus Medical Center via the European Virus Archive (EVAg). To propagate SARS-CoV-2 variants, Vero E6 cells obtained from Biomedica, Vienna, Austria (VC-FTV6), were grown until confluence in 75 cm^2^ with MEM (Gibco) and 5% FBS.

Experiments with SARS-CoV-2 were performed essentially as described in Kicker et al., 2021^26^. All working steps with the infectious SARS-CoV-2 virus were performed under BSL-3 conditions^27^.

### Virus adsorption onto PCT

Adsorption of HCoV-229E was performed by incubating the respective virus suspension in the respective serum-free culture media with PCT in a final volume of 1 ml at the indicated concentrations for 1 h at 35 °C. After pelleting PCT by centrifugation for 10 min at 4000 × g, the supernatant was collected for use in a plaque assay (HCoV-229E) or in a inactivation assay (SARS-CoV-2).

### HCoV-229E virus titration by plaque assay

MRC-5 cells were seeded onto 12-well plates until they reached 90% confluence. The cells were infected by replacing the culture medium with 500 µl of the supernatant obtained from the adsorption experiments. Supernatants were diluted beforehand with EMEM supplemented with penicillin and streptomycin when necessary, in order to reach a plaque count between 5 and 200. After 2 h incubation of the cells with the virus at RT, the virus suspension was replaced by 500 µl of overlay medium consisting of 2% methylcellulose (Alfa Aesar, Kandel, Germany) in growth medium containing all supplements (see above). After 30 min of incubation at RT, 1.5 ml of growth medium containing all supplements were added to the infected cells and the plates were further incubated at 35 °C for 24 h.

Detection of the infected plaques was performed by immunofluorescence. Upon the 24 h infection time, the cells were briefly washed with PBS (PAN-Biotech, Aidenbach, Germany) and fixed with a 1:1 mixture of methanol and acetone (both Merck KGaA, Darmstadt, Germany) for 10 min at −20 °C. After 3 further washes with PBS, the cells were incubated with PBS supplemented with 5% FBS for 1 h at RT. Labeling was performed by incubating the cells for 1 h at 37 °C with a rabbit polyclonal anti-HCoV-229E nucleocapsid antibody (SinoBiological, Beijing, China) diluted 1:400 in PBS supplemented with 1% FBS and 0.01% Triton X-100 (VWR, Darmstadt, Germany). After 3 washes with PBS, a goat anti-rabbit antibody conjugated with Alexa Fluor 488 (Thermo Fisher Sc., Schwerte, Germany) was added at a 1:400 dilution in PBS supplemented with 1% FBS and 0.01% Triton X-100 and incubated for 1 h at 37 °C. After 3 washes in PBS, the labeling was observed and plaques were counted manually using an IX83 fluorescence microscope (Olympus, Hamburg, Germany). 2 wells were counted for each condition. The virus titer was determined using the formula: No. of plaques/(dilution factor x volume of inoculum in ml). The results were expressed as plaque-forming units (PFU) per ml.

### SARS-CoV-2 inactivation assay

Vero E6 cells (3 × 10^4^ cells/well in 2% FBS MEM) were seeded into 48 well plates 24 h prior to infection under BSL 2 conditions. PCT was sterilized with 70% ethanol as described above. The virus stock was diluted with MEM to 87 PFU/well for infection. For each PCT concentration, 24.5 µL of virus dilution were mixed with 675.5 µL serum free cell culture medium (MEM) and pre-incubated with 700 µL of the PCT suspension in a reaction tube under continuous shaking for 1 h at 37 °C. After the pre-incubation time, PCT was separated from the liquid phase by centrifugation at 13,000 × g for 5 min. From the supernatant, 140 µL were collected to determine by RT-qPCR the amount of virus used for the infection (virus input). From the remaining supernatant, 200 µL were used to infect Vero E6 cells that were then incubated for 60 min at 37 °C with 5% CO_2_. Thereafter, cells were washed once with serum-free MEM and then 440 µL fresh pre-warmed cell culture medium (MEM containing 2% FBS) was added to each well. After 10 min of incubation at RT, 140 µL from the cell culture medium were collected and stored at -80 °C. Those samples were used to determine the starting concentration of viral copy numbers at t = 0 h (after washing) by RT-qPCR. At that time point, we measured constant Ct-values around 27 after one washing step with serum-free MEM, independently from the virus load applied to the cells; Therefore, Ct 27 was the background after infection and Ct values of 27 or higher indicated no virus infection and replication (t = 0 values were omitted from further experimental series). After 48 h incubation at 37 °C and 5% CO_2_, a further 140 µL of cell culture medium were collected (t = 48 h) and RNA was isolated to determine virus copy numbers by RT-qPCR. As a positive control for virus inactivation, human convalescent serum (approved by the research ethics committee of the Medical University Graz; number: 1009/2022) was added to a virus suspension instead of PCT pre-treatment. As a further control to verify loss of virus during the manipulations, a viral suspension was incubated with medium without any substance as positive control for cell infection. A negative control made of medium without virus was also included. Three replicates were used for each PCT concentration and this in turn was used to infect 3 wells each.

### Determination of virus concentration by RT-qPCR

Viral RNA was isolated from cell culture medium supernatants by using QIAamp® Viral RNA Mini Kit, as recommended by the CDC. To detect the viral load in the samples, the RT-qPCR was performed based on the CDC recommendation using QuantiTect Multiplex RT-PCR Kit with a Rotor Gene Q cycler using the following primer pairs^40^:

2019-nCoV_N2-F 2019-nCoV_N2 Forward Primer 5′-TTA CAA ACA TTG GCC GCA AA-3´

2019-nCoV_N2-R 2019-nCoV_N2 Reverse Primer 5′-GCG CGA CAT TCC GAA GAA-3′

2019-nCoV_N2-P 2019-nCoV_N2 Probe 5′-FAM-ACA ATT TGC/ZEN/CCC CAG CGC TTC AG-3IABkFQ-3′ FAM, BHQ-1

Virus replication was assessed in cell culture supernatants by comparing Ct values at the time point of infection with Ct values after different time-periods of culturing (allowing the virus to replicate). By comparing Ct values of cells infected with viral suspension without substance (positive controls for maximal virus replication) with Ct values of cells incubated with PCT-treated viral suspension at the end of the cultivation period (e.g., 48 h), an inhibitory effect on virus replication can be calculated. Cells cultivated without any virus were used to determine RT-qPCR background values. All Ct values higher than 40 were considered not detectable (nd). To calculate viral copy numbers based on the RT-qPCR Ct values a calibration curve (Figs. S1 and S2) based on a certified RNA standard (ATCC VR-1986D™) was used. This standard contains 4.73 × 10^3^ genome copies per 1 µL. Viral copy numbers of virus input, t = 0 h and t = 48 h, were calculated using the resulting equation y = 1.422x + 35.079. Potential inhibitory effects of PCT on the RT-qPCR reaction were tested by running an internal control with different PCT concentrations extracts. No interference of PCT extracts on the RT-qPCR reaction was observed.

### Virus binding demonstration by scanning electron microscopy

100 µl of a HCoV-2229E virus suspension were fixed for 20 min at RT with 4% paraformaldehyde in 50 mM Hepes Buffer at pH 7.2 and incubated with 2 µl 0.1% PCT suspension in MilliQ water for 1 h at RT. The mix was filtered onto 0.1 µm Nuclepore polycarbonate track etch membrane filters (SPI Supplies, West Chester, USA), washed 5 × with 0.1% Triton X-100 in MilliQ water and thereafter 5 × with MilliQ water. Upon air drying, the samples were fixed onto a self-made brass sample holder and coated for 15 s with a gold/palladium target (Cressington sputter coater 108).

Virus suspension was replaced with culture medium alone in negative control. Images were taken on a JEOL JSM-IT800 SEM at 5 kV using the secondary electron detector and didn’t undergo any processing.

### Detection of infected cells by immunohistochemistry

Infected cells from the inactivation assay were immunostained in 48 well plates using an anti-SARS-CoV-2 nucleocapsid antibody (SARS-CoV-2 (2019-nCoV) nucleocapsid Antibody, Rabbit Mab, Sinobiological Cat: 40143-R019). To this end, cells were fixed with 4% phosphate-buffered formaldehyde under BSL 3 conditions and washed three times with PBS. Once fixed, the cells were handled under BSL 2 conditions. The cells were permeabilized with 0.1% Triton × 100 in PBS for 10 min. After washing thrice with PBS the endogenous peroxidase was blocked with 3% H_2_O_2_ in methanol for 30 min. Then the cells were incubated with the primary anti-SARS-CoV-2 nucleocapsid antibody in antibody diluent (REAL Antibody diluent, Agilent Technologies, Dako Cat: S202230_2). After three washing steps with PBS, cells were incubated with the secondary antibody (EnVision™ + Dual Link System HRP, Agilent Technologies, Dako Cat: K5007) for 30 min. After three washing steps with PBS, the substrate (AEC substrate-chromogen, Agilent Technologies, Dako, Cat: K346430-2, 2 drops) was applied on the cells and incubated until viral infected cells were stained red, but not longer than 3 min. Reaction was stopped by washing three times in PBS and wells were kept humid until photo documentation. One picture of a representative area of the well was chosen per condition (Fig. 5) using a Nikon Eclipse TS100 microscope with the software of Jenoptik “GRYPHAX”.

### Metabolic interference assay

For analysis of metabolic interference of the PCT concentrations used for virus inactivation assay, a resazurin assay was performed. 24 h prior to the metabolic interference assay Vero E6 cells were seeded with a density of 3 × 10^4^ cells per well in three 48-well cell culture plates in MEM (Gibco) medium supplemented with 2% FBS, 2% L-glutamine and 1% penicillin/streptomycin. On the first day of measurement (t = 0), PCT was weighted and sterilized with 70% ethanol as described above. The resulting suspension at 0.1%, 0.4%, 2% and 10% was incubated for 1 h at RT under continuous shaking to simulate the incubation time with the virus. The supernatant was collected after centrifugation at 2000 × g for 10 min. The culture medium of the Vero E6 cells was removed and replaced with 200 µL of those supernatants for 1 h at 37 °C and 5% CO_2_. Afterwards, the cells were washed once with serum-free MEM and fresh, pre-warmed MEM with 2% FBS was applied to the cells (starting point t = 0 h). At the time points 0 h, 24 h and 48 h, 10 µM resazurin was added and fluorescence signals were measured at Ex485/Em590 nm over 2 h incubation of the cells with resazurin. The Δk of the fluorescence signals (slope) correlates with the metabolic activity.

### Statistical analysis

Statistical analysis was performed using Graphpad Prism 9. Significance was determined using mean rank comparison (for two groups Mann-Whitney test, for more than two groups Kruskal-Wallis test: comparing the mean rank of control column (no substance) with the mean rank of every other column in the graph). Significance for *p*-values is defined as : *p* > 0.05 (not significant; ns); *p* ≤ 0.05 (*); *p* ≤ 0.01 (**); *p* ≤ 0.001 (***)^41^.

## Supplementary Information

Supplementary Information.

## Data Availability

The datasets generated and/or analyzed during the current study are available from the corresponding author on reasonable request.
