# Supplementary material for: Binding and inactivation of human coronaviruses, including SARS-CoV-2, onto purified clinoptilolite-tuff
Source: Sci Rep. 2023 Mar 22;13:4673. doi: 10.1038/s41598-023-31744-z (PMC10031168; doi:10.1038/s41598-023-31744-z)
Supplement: Supplementary file 1 — Supplementary Information. [file 41598_2023_31744_MOESM1_ESM.docx]

Supplementary information

**S1 and S2: Calculation of the reduction in virus copy numbers in neutralization assays**

The virus copy numbers were calculated from Ct values of the experiments by using a certified virus SARS-CoV-2 RNA standard. By using serial dilutions of the reference RNA for the RT-qPCR (measured in duplicates), a standard curve (Fig S1) can be calculated. With the defined containing genome copies and the related linear equation, an efficiency of 78.28% (Fig S2) was determined for the used primer pairs and RT-qPCR machine.


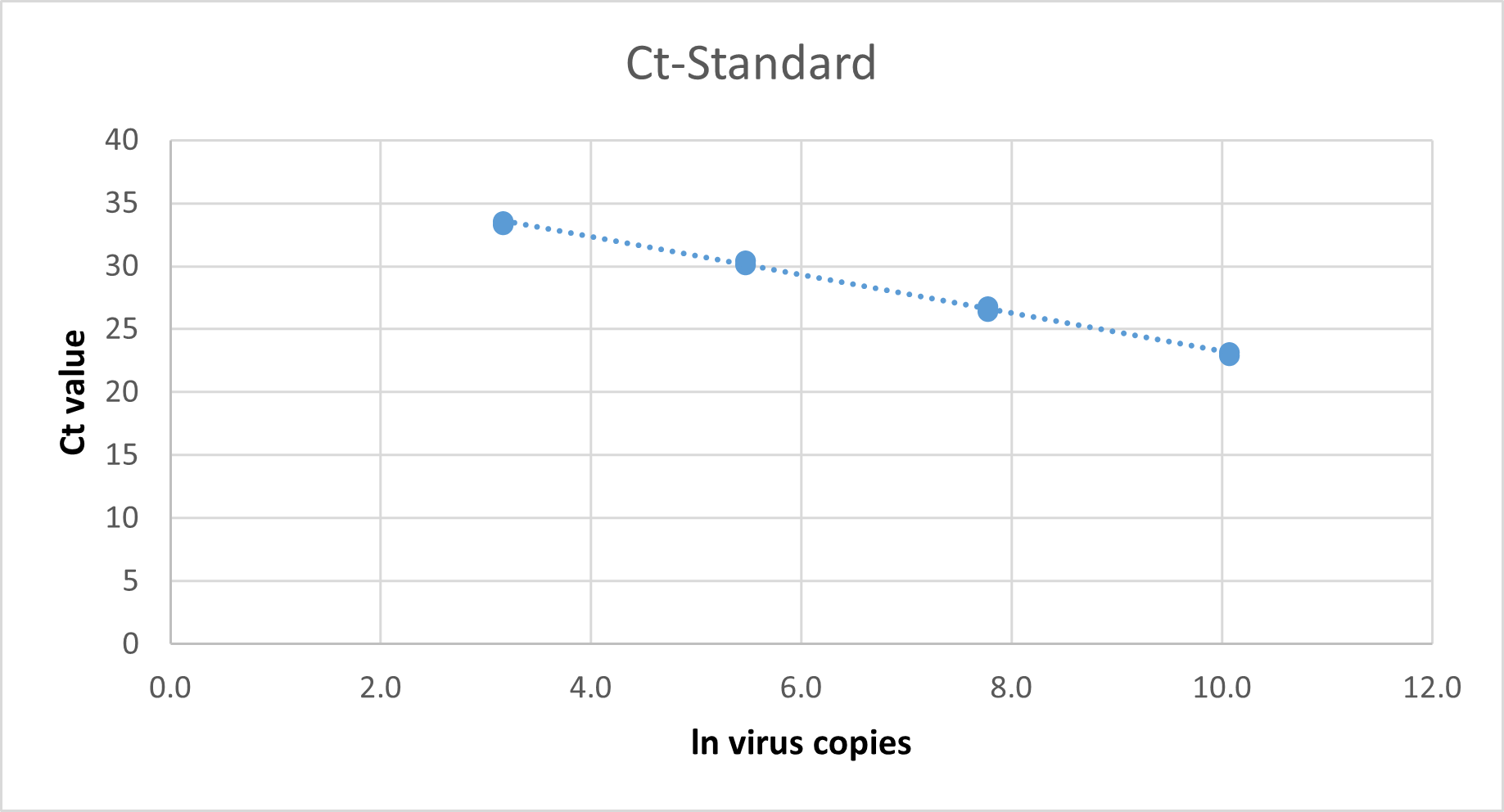


**Figure S1**: calculation of the standard curve. Slope: $y= -1.51x+38.357$


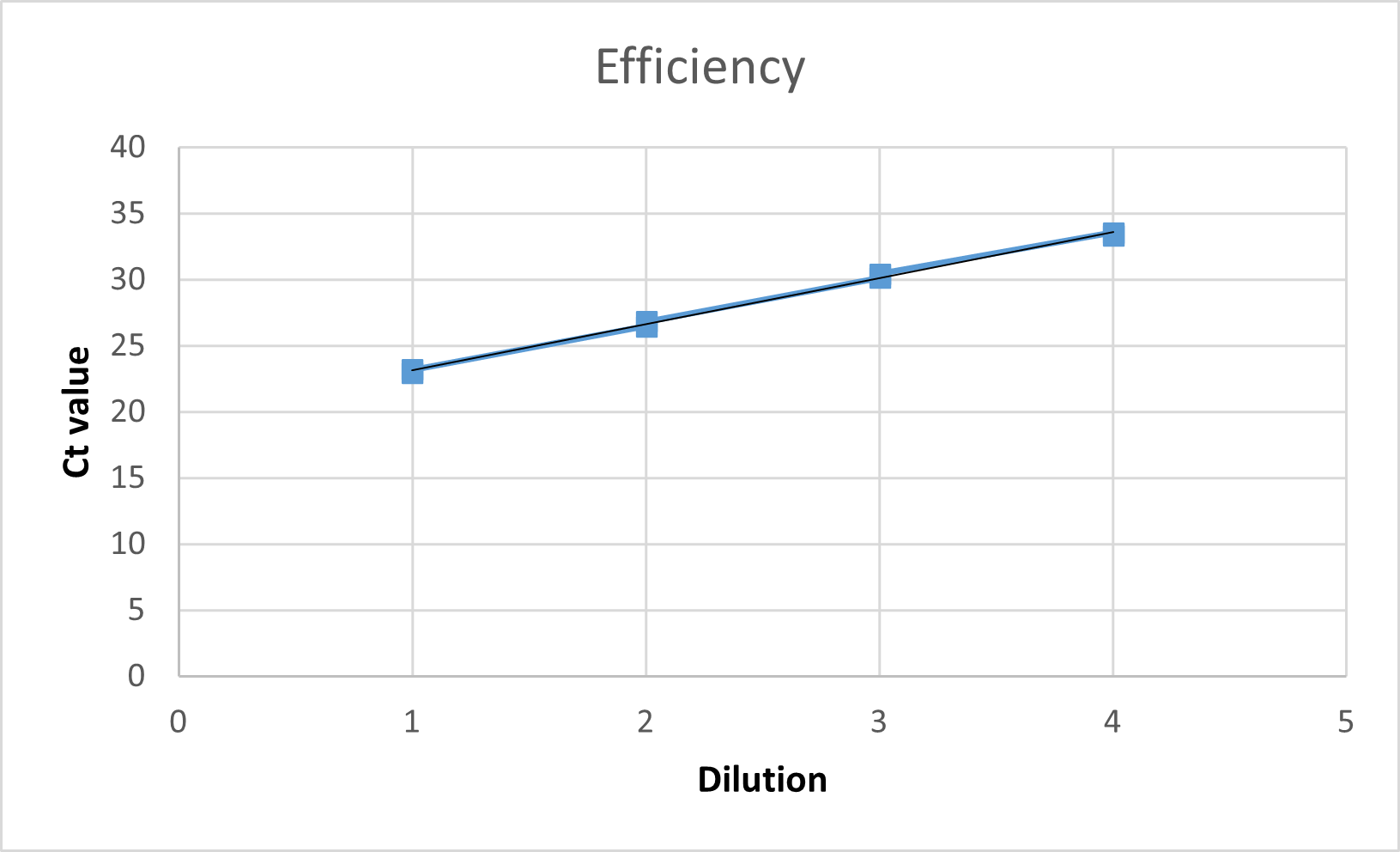


**Figure S2**: RT-qPCR efficiency. Slope: $y= 3.477+19.673$
